# Honokiol Enhances Paclitaxel Efficacy in Multi-Drug Resistant Human Cancer Model through the Induction of Apoptosis

**DOI:** 10.1371/journal.pone.0086369

**Published:** 2014-02-25

**Authors:** Xu Wang, Jonathan J. Beitler, Hong Wang, Michael J. Lee, Wen Huang, Lydia Koenig, Sreenivas Nannapaneni, A. R. M. Ruhul Amin, Michael Bonner, Hyung Ju C. Shin, Zhuo Georgia Chen, Jack L. Arbiser, Dong M. Shin

**Affiliations:** 1 Department of Hematology and Medical Oncology, Winship Cancer Institute, Emory University School of Medicine, Atlanta, Georgia, United States of America; 2 Department of Radiation Oncology and Otolaryngology, Winship Cancer Institute, Emory University School of Medicine, Atlanta, Georgia, United States of America; 3 Emory College of Arts and Sciences, Atlanta, Georgia, United States of America; 4 Quest Diagnostics, Atlanta, Georgia, United States of America; 5 Department of Dermatology, Winship Cancer Institute, Emory University School of Medicine, and Atlanta Veterans Administration Medical Center, Atlanta, Georgia, United States of America; Virginia Commonwealth University, United States of America

## Abstract

Resistance to chemotherapy remains a major obstacle in cancer therapy. This study aimed to evaluate the molecular mechanism and efficacy of honokiol in inducing apoptosis and enhancing paclitaxel chemotherapy in pre-clinical multi-drug resistant (MDR) cancer models, including lineage-derived human MDR (KB-8-5, KB-C1, KB-V1) and their parental drug sensitive KB-3-1 cancer cell lines. *In vitro* analyses demonstrated that honokiol effectively inhibited proliferation in KB-3-1 cells and the MDR derivatives (IC_50_ ranging 3.35±0.13 µg/ml to 2.77±0.22 µg/ml), despite their significant differences in response to paclitaxel (IC_50_ ranging 1.66±0.09 ng/ml to 6560.9±439.52 ng/ml). Honokiol induced mitochondria-dependent and death receptor-mediated apoptosis in MDR KB cells, which was associated with inhibition of EGFR-STAT3 signaling and downregulation of STAT3 target genes. Combined treatment with honokiol and paclitaxel synergistically augmented cytotoxicity in MDR KB cells, compared with treatment with either agent alone *in vitro*. Importantly, the combined treatment significantly inhibited *in vivo* growth of KB-8-5 tumors in a subcutaneous model. Tumor tissues from the combination group displayed a significant inhibition of Ki-67 expression and an increase in TUNEL-positive cells compared with the control group. These results suggest that targeting multidrug resistance using honokiol in combination with chemotherapy drugs may provide novel therapeutic opportunities.

## Introduction

Chemotherapy remains one of major treatment options for many types of cancer, however, a significant percentage of patients develop drug resistance during the course of chemotherapy, and inevitably progress without cure [1], [2]. Despite the remarkable progress in drug development, paclitaxel with its broad anticancer spectrum has solidified the disruption of microtubule dynamics as one of the most effective anticancer strategies in use today. However, the emergence of drug-resistant cancer cells has greatly limited its clinical efficacy. It is therefore imperative to develop novel strategies to reduce or overcome chemoresistance in cancer. Various approaches to enhance chemoresponse in chemoresistant cancer models have been explored, including those combining natural products or compounds with paclitaxel or other standard chemotherapy reagents.

Honokiol is a natural component isolated from the bark of magnolia tree [2], [3], which has been traditionally used to treat anxiety-related disorders and digestive complaints [2], [4]. Interestingly, honokiol also exhibited potent anticancer activities [5], [6], [7] and further enhanced conventional chemotherapies in a variety of preclinical models of human cancer, including chronic lymphocytic leukemia [3], prostate cancer [8] and multiple myeloma [9]. These relatively wide-ranging anticancer capabilities and favorable safety profile make honokiol an attractive adjunct therapy to enhance conventional chemotherapy in clinical settings.

Overexpression of anti-apoptotic proteins is an underlying mechanism that contributes to the acquisition of therapeutic resistance, recurrence and metastasis. A growing body of evidence suggests that three anti-apoptotic proteins, i.e., survivin, Mcl-1, and Bcl-2, may be directly related to drug resistance in cancer [10], [11]. Inhibition of these crucial survival factors has been shown to trigger apoptosis and sensitize cancer cells to drug treatment [5]–[11], thus this approach may be promising in overcoming chemoresistance and improving chemotherapy in cancer.

By using a series of lineage-derived KB human squamous cancer cell lines that exhibit distinct sensitivities to paclitaxel treatment, we demonstrated that honokiol could effectively induce apoptosis in multi-drug resistant (MDR) cancer cells. Mechanistic studies showed that honokiol inhibits the EGFR-STAT3 signaling pathway, and suppresses the expression of survivin and other survival factors. We further demonstrated the *in vivo* efficacy of honokiol in treating MDR cancer and enhancing paclitaxel efficacy.

## Materials and Methods

### Cell Culture

The KB-3-1 and its MDR derivative cell lines were generously provided by Dr. Michael M. Gottesman (NCI, NIH, Bethesda, MD) and have been characterized previously [12]. KB-3-1 cells were maintained in DMEM medium supplemented with 10% fetal bovine serum. KB-8-5 and KB-C1 cells were maintained in DMEM medium supplemented with 10% fetal bovine serum and 0.01 and 1 µg/ml colchicine respectively. KB-V1 cells were maintained in DMEM medium supplemented with 10% fetal bovine serum and 1 µg/ml vinblastine. To eliminate impact of colchicine or vinblastine on experiment result, resistant cells were cultured in drug free medium for one week before any experiment.

### Cell Growth Assay

Cells were seeded at a density of 5×10^3^ cells per well into 96-well plates in quadruplet. Twenty-four hours later, drugs were added in various concentration ranges as single agents or in two-drug combinations and then incubated for 72 h. The range for honokiol was from 0.625 to 20 µg/ml for all four cell lines. For paclitaxel, the concentration range was 0.19 to 97.5 ng/ml, 3.02 ng/ml to 3.12 µg/ml, 12.1 ng/ml to 25.0 µg/ml, and 97.5 ng/ml to 100 µg/ml for KB-3-1, KB-8-5, KB-C1, and KB-V1 cells, respectively. Cell growth inhibition was measured by determining cell density with the sulforhodamine B assay. The percentage of inhibition was determined by comparison of cell density in the drug-treated cells with that of the untreated control cells. All experiments were repeated at least three times.

### Apoptosis Analysis

Apoptosis was analyzed in all four cell lines. Cells were treated with honokiol, paclitaxel, or their combination as indicated in the figure legends, trypsinized, and washed in cold 1× PBS. The cells were resuspended in 1× Annexin binding buffer (BD PharMingen), and then stained with Annexin V-phycoerythrin (Annexin V-PE; BD PharMingen) and 7-AAD (BD PharMingen) for 15 min at room temperature. The stained samples were measured using a fluorescence-activated cell sorting (FACS) caliber bench-top flow cytometer (Becton Dickinson, Franklin Lakes, NJ). FlowJo software (Tree Star, Ashland, OR) was used for apoptosis analysis. The experiments were repeated 3 times independently.

### Immunoblotting

Thirty microgram protein from whole-cell extracts or cytoplasmic and mitochondrial fractions were quantified, separated on SDS-PAGE gels and transferred to nitrocellulose membranes. After being blocked with 5% nonfat dry milk in TBS-T buffer, the membranes were incubated with specific antibodies overnight at 4°C. Mouse anti-β-actin antibody (Trevigen, Gaithersburg, MD) was used as a sample loading control. Immunostained protein bands were detected with an enhanced chemiluminescence kit (Amersham, Buckinghamshire, UK). The experiments were repeated 3 times.

### *In vivo* Anti-tumor Efficacy Assay

The animal experiment was approved by the Institutional Animal Care and Use Committee of Emory University. KB-8-5 cells (5×10^5^) were injected s.c. into 4–5 week-old female nude mice (Athymic *nu/nu*, Taconic NY). When the tumors had developed to about 100 mm^3^, the mice were divided into four groups (n = 7 or 8) in a way to minimize weight and tumor size differences among the groups: control group treated with 20% intralipid (Baxter Healthcare), honokiol group (1.0 mg/mouse or 50 mg/kg), paclitaxel group (20 mg/kg), and honokiol (1.0 mg/mouse or 50 mg/kg) plus paclitaxel (20 mg/kg) combination group. Honokiol was dissolved in 100% ethanol and mixed with 20% intralipid in a 1∶14 (v/v) ratio. Honokiol was administered to the mice 3 times per week at 1.0 mg/mouse (or 50 mg/kg) via intraperitoneal injection. Paclitaxel was administered to the mice once per week at 20 mg/kg through tail vein injection. The therapy was continued for 4 weeks. The body weight and tumor size were measured three times per week. The tumor volume was calculated using the formula: *V* = ^/^/6 × larger diameter × (smaller diameter) [13]. The mice were sacrificed 4 weeks after the initiation of treatment. Tumor and organ tissues (liver, heart, lung, spleen, and kidney) were collected for H&E staining and immunostaining analyses.

### Immunohistochemistry and TUNEL Assay

Immunohistochemical analysis for Ki-67 staining on paraffin-embedded mouse xenograft tissue was performed as previously described [13]. Cells staining positive for Ki-67 were counted and the percentage of positive cells was calculated. An average of the 8 readings was used for statistical analysis.

TUNEL assay was performed by immunofluorescence using the same specimens as above, following the procedure provided by the manufacturer (Promega, Madison, WI). To analyze the assay results, the total cell number and the positive cell number in the same area were counted for five random areas; the result was presented as an average ratio of positive cell number out of the total cell number.

### Statistical Analysis

The statistical significance of treatment of cells in the *in vitro* cytotoxicity assay was assessed using the Student’s *t*-test. For *in vivo* anti-tumor efficacy assay, a log-linear mixed model with random intercept was used to compare the significance of the mean tumor volumes among each group. The statistical significance of treatment effect on microtubules, apoptosis, and cell proliferation in xenograft tumor tissues was assessed using the Kruskal-Wallis test (one-way ANOVA). *P*<0.05 was considered statistically significant in all analyses.

## Results

### Honokiol Reduces Viability and Induces Apoptosis in Human MDR Cancer Cells

**Figure 1:**
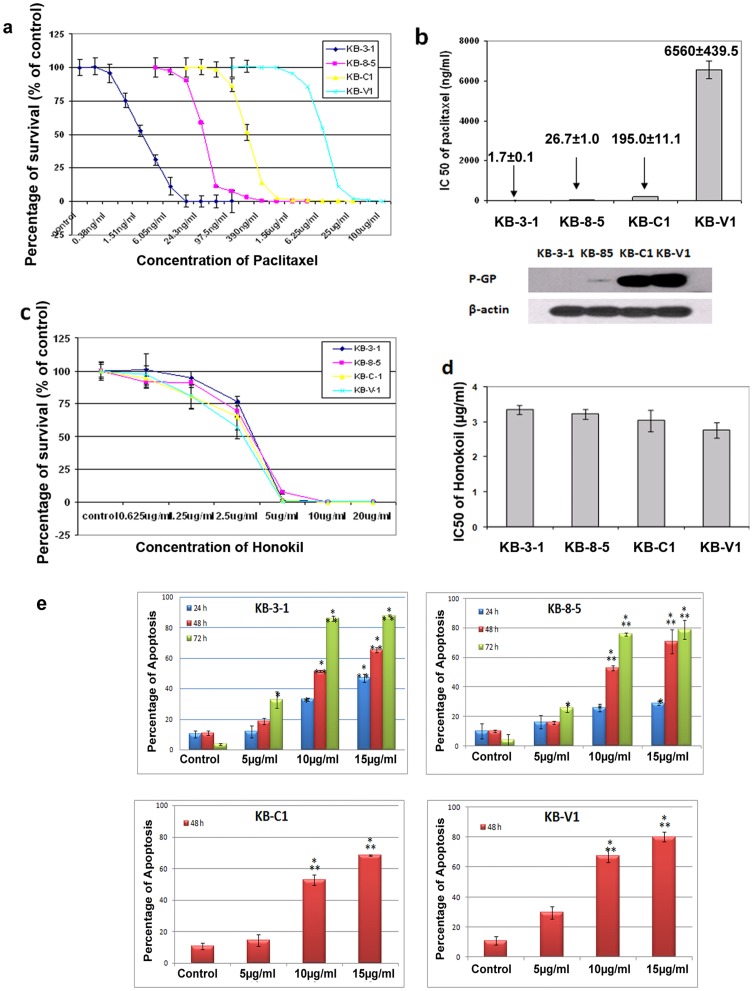
Honokiol inhibits growth and induces apoptosis in multidrug-sensitive and -resistant cells. (**a, b**) Growth inhibition effect of paclitaxel and p-gp expression in KB-3-1, KB-8-5, KB-C1 and KB-V1 cells. The IC_50_ values of paclitaxel in drug resistant KB-8-5, KB-C1 and KB-V1 cells were increased by 16-, 117-, and 4000-fold respectively when compared to parental KB-3-1 cells. (**c, d**) Growth inhibition effect of honokiol in KB-3-1, KB-8-5, KB-C1 and KB-V1 cells. The IC_50_ values of honokiol in drug resistant KB-8-5, KB-C1 and KB-V1 cells were similar to that in parental KB-3-1 cells. (**e**) Honokiol induces apoptosis in KB cells. KB-3-1 and KB-8-5 cells were treated with 5, 10, 15 µg/ml of honokiol for 24, 48, and 72 h. KB-C1 and KB-V1 cells were treated with honokiol for 48 h. Apoptosis was measured by annexin V-phycoerythrin staining. *indicates statistically significant p values (*,versus control, p<0.05; **, versus 5 µg/ml, p<0.05).

The MDR cancer cell lines KB-8-5, KB-C1 and KB-V1 were derived from their drug-sensitive counterpart KB-3-1 cells, and have been widely used as a clinically relevant model in the study of drug resistance [14], [15]. These cells exhibited distinct sensitivities to paclitaxel treatment. As shown in **Fig. 1a, 1b**, the IC_50_ values in MDR cell lines (KB-8-5∶26.73±1.01 ng/ml, KB-C1∶195.0±11.11 ng/ml, and KB-V1∶6560.0±439.52 ng/ml) were increased by 16-, 117- and 4,000-fold, respectively, when compared to the parental KB-3-1 cell line (1.66±0.09 ng/ml). Consistent with their resistant phenotype, MDR cell lines substantially express the classical MDR marker P-glycoprotien (**Fig. 1b** **)**. Interestingly, a 72 h-treatment with honokiol effectively reduced the viability of MDR cells (KB-8-5∶3.22±0.14 µg/ml, KB-C1∶3.04±0.30 µg/ml, KB-V1∶2.77±0.22 µg/ml), with IC_50_ values similar to those in KB-3-1 cells (3.35±0.13 µg/ml) (**Fig. 1c, 1d**). Apoptosis analysis further showed that honokiol induced apoptosis in a time- and dose-dependent manner (**Fig. 1e** **).** 24 h following honokiol treatment, the percentage of apoptotic KB-3-1 cells was 11.9±3.9% (5 µg/ml of honokiol), 32.9±0.9% (10 µg/ml), and 47.1±2.7% (15 µg/ml); the percentage of apoptotic cells increased to 18.4±2.1%, 51.5±0.6%, and 65.3±1.6%, respectively, following a 48 h-treatment, which was further increased at 72 h. Honokiol also induced a similar degree of apoptosis in other MDR cells, including KB-8-5, KB-C1 and KB-V1 (**Fig. 1e** **)**. For example, a 48-h treatment with 10 µg/ml of honokiol resulted in apoptosis in 51.5% of KB-3-1 cells, 52.7% of KB-8-5 cells, 52.9% of KB-C1 cells and 67.6% of KB-V1 cells, respectively. These data are consistent with the viability assay (**Fig. 1c, 1d**), and suggest that honokiol is a potent cytotoxic agent in KB cells regardless of their different paclitaxel sensitivities.

### Honokiol Induces Mitochondria- and Death Receptor (DR)-dependent Apoptosis in Human Cancer Cells

**Figure 2:**
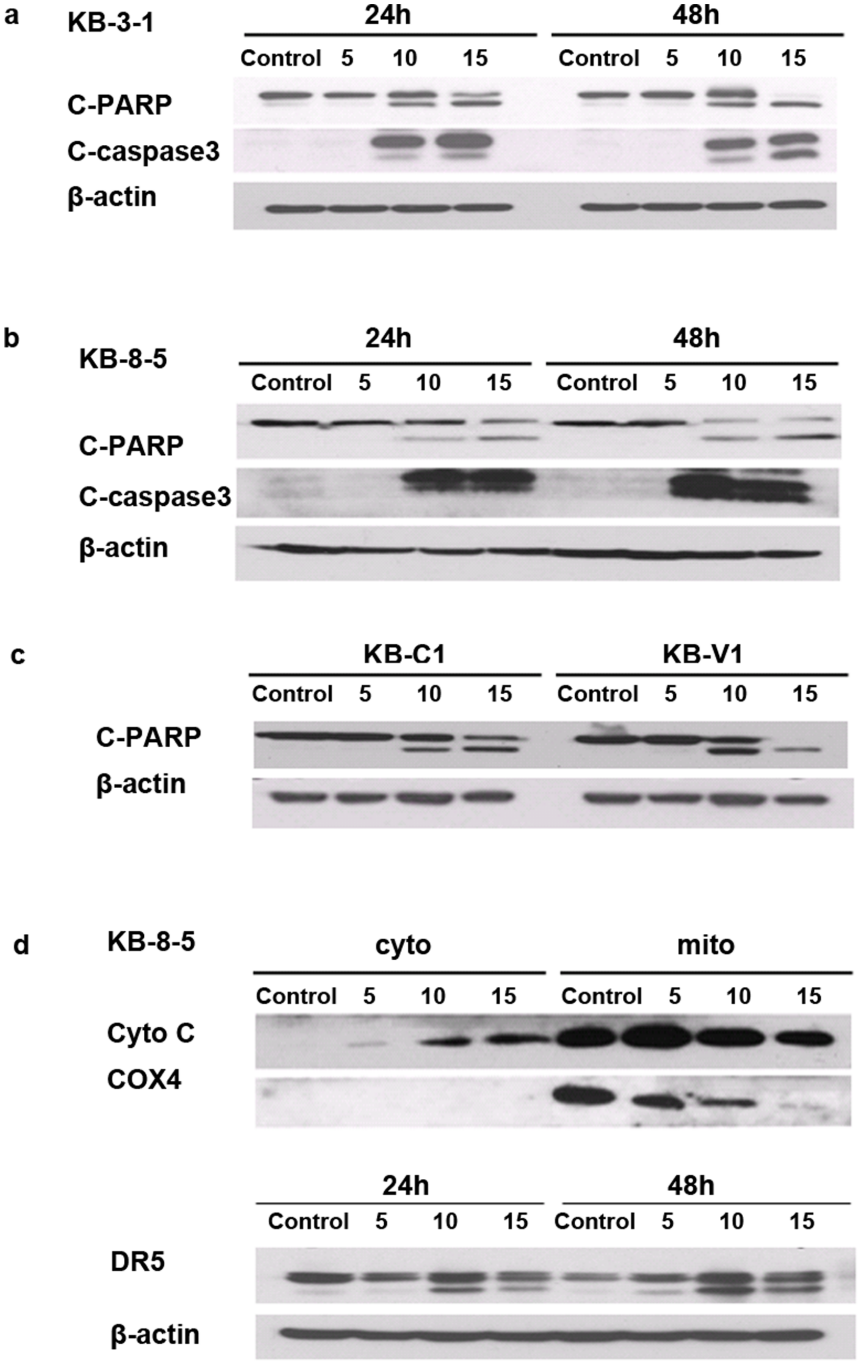
Honokiol induces PARP and caspase-3 cleavage and the release of cytochrome *c* in the cytoplasm in multidrug-sensitive and -resistant cells. (**a**) KB-3-1 cells and (**b**) KB-8-5 cells were treated with 5, 10, 15 µg/ml of honokiol for 24, 48 h. Full-length and cleaved PARP, cleaved caspase-3 and β-actin as a loading control were detected by immunoblotting using whole cell lysates. (**c**) KB-C1 and KB-V1 cells were treated with 5, 10, 15 µg/ml of honokiol for 48 h. Cleaved PARP was detected by immunoblotting using whole cell lysates. (**d**) Upper panel, KB-8-5 cells were treated with 5, 10, 15 µg/ml of honokiol for 48 h. Cytoplasmic and mitochondrial fractions were separated and immunoblotted with cytochrome c (Cyto C) antibody. COX4 (a mitochondrial protein) was used to show efficiency of cell fractionation. Lower panel, KB-8-5 cells were treated with 5, 10, 15 µg/ml of honokiol for 24 and 48 h. DR5 was detected by immunoblotting using whole cell lysates. All experiments were repeated at least three times, and representative data are presented.

We investigated the mechanism of honokiol-induced apoptosis in KB cells. As shown in **Fig. 2a–c** **,** honokiol treatment induced the cleavage of PARP and caspase-3 in a dose- and time-dependent manner in all the tested KB cells, indicating the activation of apoptotic signals. Western blot analyses found that honokiol not only induced the release of cytochrome *c* in the cytoplasm in a dose-dependent manner **(** **Fig. 2d** **)**, but also increased the expression of DR5 (**Fig. 2d**), a surface receptor for pro-apoptotic ligands Apo2L/TRAIL. Taken together, these data suggest that honokiol could simultaneously activate mitochondria-dependent (intrinsic) apoptosis and DR-dependent (extrinsic) apoptosis in KB cells.

### Honokiol Inhibits EGFR-STAT3 Signaling in Human Cancer Cells

**Figure 3:**
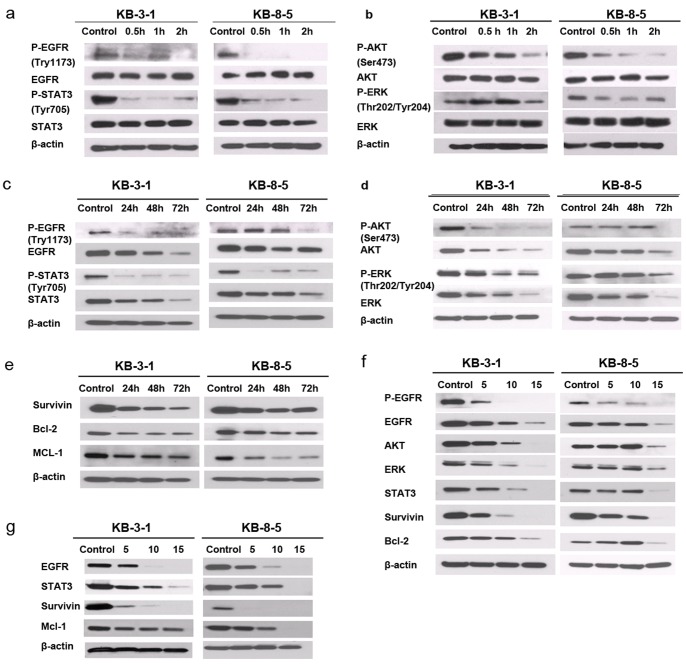
Honokiol inhibits the EGFR signaling pathway and downregulates STAT3 target genes in multidrug-sensitive and -resistant cells. KB-3-1 and KB-8-5 cells were treated with 5 µg/ml of honokiol for short time (0.5, 1, or 2 h) or long time (24, 48, or 72 h). Whole cell lysates were immunoblotted for the indicated proteins. The effect of honokiol at early time points: (**a**) phosphorylation of EGFR and STAT3; (**b**) Honokiol decreases phosphorylation of AKT and ERK. The effect of honokiol at late time points: (**c**) phosphorylation and expression of EGFR and STAT3; (**d**) phosphorylation and expression of AKT and ERK; (**e**) expression of survivin, Bcl-2, Mcl-1. (**f**) Honokiol inhibits the EGFR signaling pathway and downregulates STAT3 target genes in a dose-dependent manner. KB-3-1 and KB-8-5 cells were treated with 5, 10, 15 µg/ml of honokiol for 48 h. Whole cell lysates were immunoblotted for the indicated proteins. (**g**) Honokiol inhibits the EGFR-STAT3 pathway in KB-C1 and KB-V1 cells. KB-C1 and KB-V1 cells were treated with 5, 10, 15 µg/ml of honokiol for 48 h. All experiments were repeated at least three times, and representative data are presented.

The EGFR-STAT3 signaling pathway plays an important role in the regulation of growth and survival in cancer cells. We investigated the effects of honokiol on several key components of the EGFR-STAT3 signaling pathway. KB-3-1 and KB-8-5 cells were treated with honokiol (5 µg/ml) for 0.5, 1, and 2 h. Western blot analysis using total cell lysates revealed a markedly reduced phosphorylation of EGFR at Try1173, an indicator of activated EGFR (**Fig. 3a**). We further examined the phosphorylation status of STAT3 (Tyr705), Akt (Ser473), and ERK (Thr202/Tyr204), three known EGFR downstream signaling components. Interestingly, honokiol treatment rapidly inhibited phosphorlylation of STAT3 and AKT, but had no effect on ERK phosphorylation (**Fig. 3ab**). To further examine whether the expression level of EGFR and its downstream target proteins are also inhibited by honokiol treatment (5 µg/ml), we treated cells for 24, 48, and 72 h. As shown in **Fig. 3cd**, honokiol treatment inhibited protein expression of EGFR, STAT3, ERK, and AKT in a time-dependent manner in KB-3-1 and KB-8-5 cells. Similarly, phosphorylation of those proteins was decreased upon honokiol treatment (**Fig. 3cd)**. Consistently, the expression levels of three known STAT3 target genes, i.e., survivin, Bcl-2 and Mcl-1, were dramatically decreased following honokiol treatment (**Fig. 3e** **)**.

We next examined the dose-dependent response of EGFR-STAT3 signaling to honokiol treatment in KB-3-1 and KB-8-5 cells. As shown in **Fig. 3f**, the expression and phosphorylation of EGFR (Try1173) were decreased in the presence of 5 or 10 µg/ml of honokiol (48 h), and were further decreased at a dose of 15 µg/ml of honokiol (**Fig. 3f** **)**. Consistently, the expression and/or phosphorylation of EGFR downstream signaling components, including ERK, Akt, STAT3, survivin, Bcl-2 and Mcl-1, were also dramatically inhibited in a dose-dependent manner (**Fig. 3f** **)**. A similar effect of honokiol on EGFR-STAT3 signaling was also observed in both KB-C1 and KB-V1 cells (**Fig. 3g** **)**. Of particular interest, an RT-PCR analysis found that honokiol inhibition of survivin expression may involve the suppression of survivin transcription at the mRNA level (**Figure S1)**.

### Honokiol Enhances the in vitro Cytotoxicity of Paclitaxel in MDR Cancer Cells

**Figure 4:**
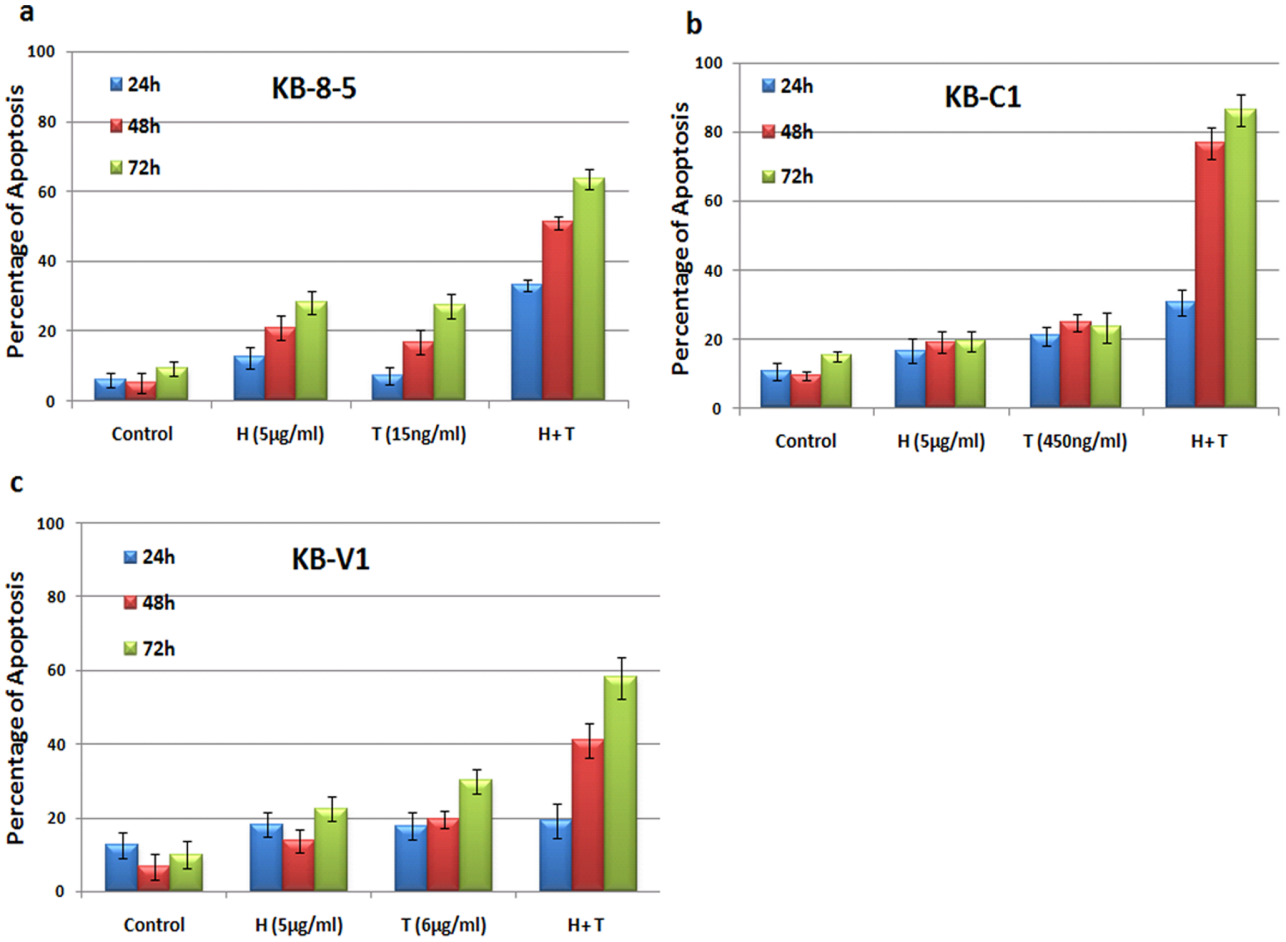
Synergistic apoptosis induced by the combination of honokiol and paclitaxel in multidrug-resistant cells. (**a**) KB-8-5, (**b**) KB-C1, and (**c**) KB-V1 cells were treated with 5 µg/ml of honokiol, paclitaxel (15 ng/ml for KB-8-5, 450 ng/ml for KB-C1, and 6 µg/ml for KB-V1), or the combination of two drugs at the indicated concentrations for 24, 48, and 72 h. Apoptosis was measured. *indicates statistically significant p values (*, combination versus honokiol, p<0.05; **, combination versus paclitaxel, p<0.05). All experiments were repeated three times.

We investigated whether the inhibition of survivin, Bcl-2 and Mcl-1 by honokiol could sensitize MDR cancer cells to conventional chemotherapy such as palictaxel. As shown in **Fig. 4**, 24-h treatment with either honokiol (5 µg/ml) or paclitaxel (450 ng/ml) resulted in ≤20% of apoptosis in KB-C1 cells, whereas combined treatment using both honokiol and paclitaxel resulted in approximately 30% apoptosis. Moreover, at the 48 h and 72 h time points, the combined treatment more effectively induced apoptosis (76% and 86%, respectively) in KB-C1 cells than either single agent (≤25%) or the vehicle control. Consistently, the combined treatment was more effective in inducing apoptosis than either single agent in MDR KB-8-5 and KB-V1 cells (**Fig. 4**). Using KB-8-5 cells as an example, a combination index (CI) assay confirmed the synergistic effect of combined treatment with honokiol and paclitaxel in reducing the viability of MDR cancer cells (**Table S1**).

**Figure 5:**
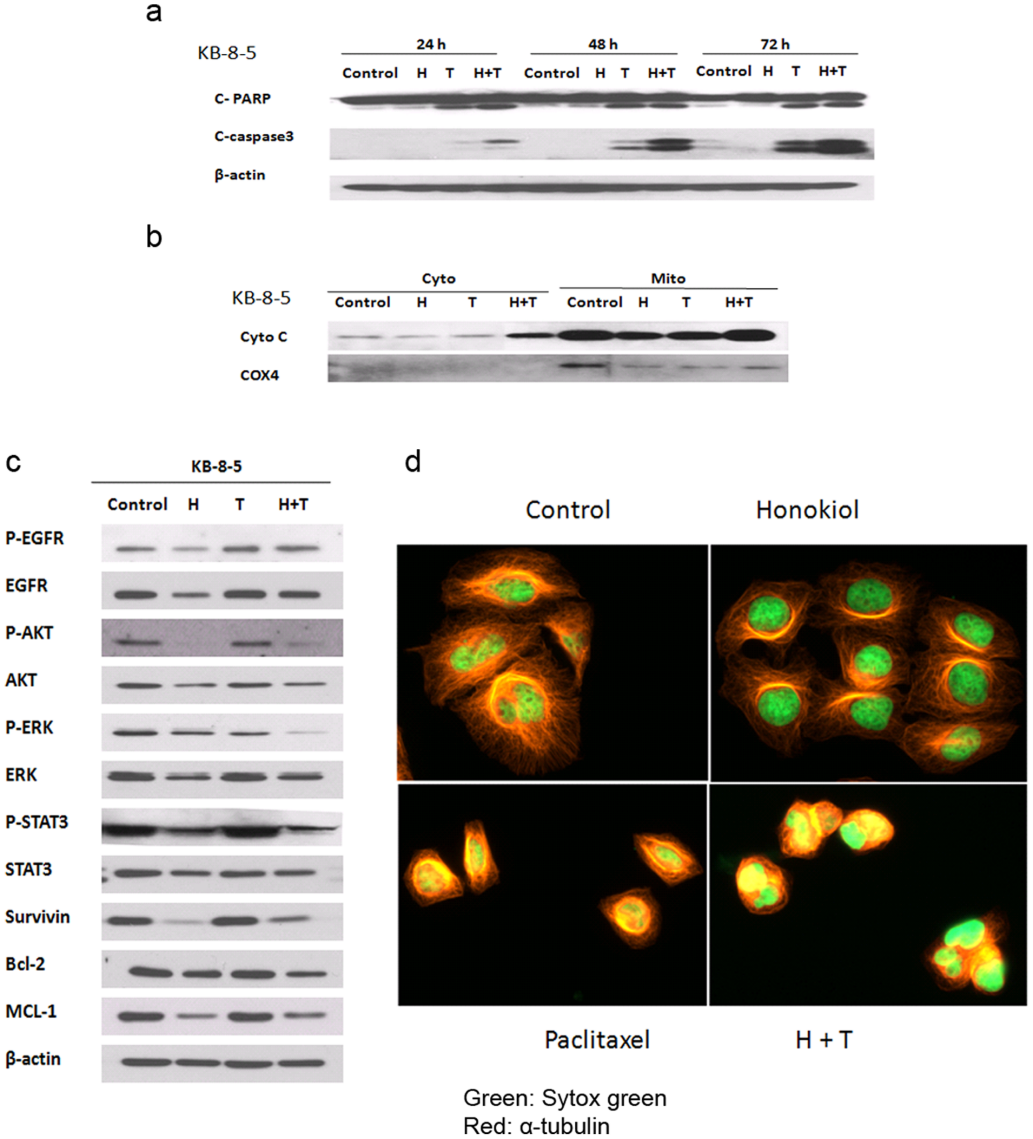
Honokiol enhancement of paclitaxel induced apoptosis involves the inhibition of the EGFR-STAT3 signaling pathway. (**a**) Honokiol enhances paclitaxel induced PARP and caspase-3 cleavage in KB-8-5 cells. The cells were treated with 5 µg/ml of honokiol, 15 ng/ml of paclitaxel, or the combination of the two drugs for 24, 48, and 72 h. Full-length and cleaved PARP, cleaved caspase-3 and β-actin as a loading control were detected by immunoblotting using whole cell lysates. (**b**) Honokiol enhances paclitaxel induced release of cytochrome *c* in the cytoplasm in KB-8-5 cells. The cells were treated with 5 µg/ml of honokiol, 15 ng/ml of paclitaxel, or the combination for 48 h. Cytoplasmic and mitochondrial fractions were separated and immunoblotted with cytochrome c (Cyto C) antibody. COX4 (a mitochondrial protein) was used to show efficiency of cell fractionation. (**c**) Honokiol inhibits the EGFR-STAT3 signaling pathway and downregulates STAT3 target gene expression in KB-8-5 cells. The cells were treated with 5 µg/ml of honokiol, 15 ng/ml of paclitaxel, or the combination for 48 h. Whole cell lysates were immunoblotted for the indicated proteins. (**d**) Paclitaxel induces microtubule polymerization in KB-8-5 cells. Drug-induced stabilization of microtubules as evidenced by an increase in microtubule polymer mass resulting in bundling was observed upon treatment with both paclitaxel and the combination; however, honokiol alone was not effective in microtubule stabilization (magnification 400x). The cells were treated with 5 µg/ml of honokiol, 15 ng/ml of paclitaxel, or the combination for 24 h. All experiments were repeated at least three times, and representative data are presented.

Western blot analysis further found that the combined treatment with honokiol and paclitaxel was more effective than either agent alone in inducing the cleavage of caspase-3, PARP, and the release of cytochrome *c* from mitochondria in KB-8-5 cells (**Fig. 5a, 5b**). A 48-h treatment with honokiol alone or the combination of honokiol and paclitaxel decreased the expression and phosphorylation of EGFR, as well as other key EGFR-STAT3 signaling components, including STAT3, p-STAT3(Tyr705), ERK, p-ERK(Thr202/Tyr204), Akt and p-Akt(Ser473). The protein expression of survivin, Bcl-2 and Mcl-1 was also markedly reduced upon the treatment with honokiol or the combination of honokiol and paclitaxel. Interestingly, however, the treatment with paclitaxel alone did not have significant effect on these signaling components or target genes of the EGFR-STAT3 pathway (**Fig. 5c**).

Paclitaxel exerts its antitumor activity by binding to tubulin inside the lumen of the microtubule, resulting in microtubule polymerization, stabilization and disruption of microtubule dynamics, causing mitotic arrest and apoptosis in proliferating cells. To investigate whether honokiol can enhance paclitaxel induced microtubule polymerization and stabilization, we examined the effect of each treatment on microtubules in KB-8-5 and KB-C1 cells. Drug-induced stabilization of the tumor cells’ interphase microtubules, as evidenced by an increase in microtubule polymer mass resulting in bundling, was observed upon treatment with both paclitaxel and the combination; however, honokiol alone was not effective in the formation of microtubule bundles **(** **Fig. 5d** **).** Collectively, these data indicate that honokiol enhances the *in vitro* cytotoxicity of paclitaxel in MDR cancers, may be through the inhibition of EGFR-STAT3 survival signaling.

### Honokiol Enhances the in vivo Efficacy of Paclitaxel in MDR Cancer Xenograft Tumors

**Figure 6:**
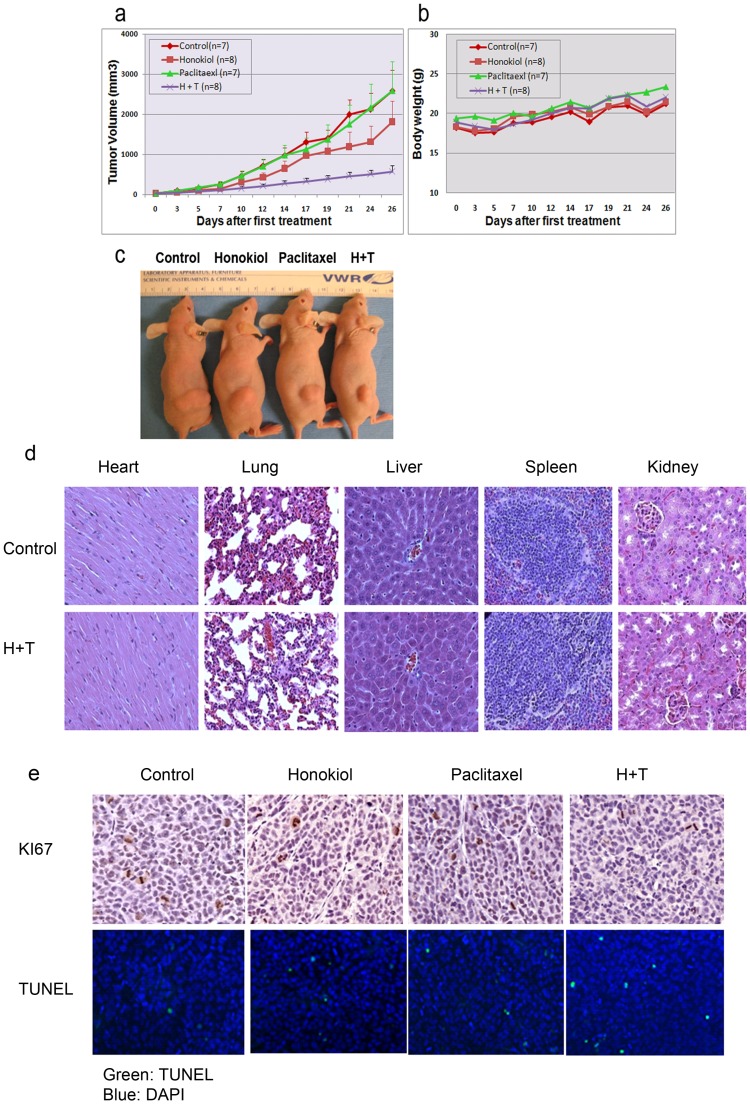
Inhibition of tumor growth by the combination of honokiol and paclitaxel in a multidrug-resistant xenograft model. (**a**) The tumor growth of KB-8-5 xenografts was significantly inhibited in the combination-treated group compared with the control (p<0.0001), honokiol (p = 0.0356) and paclitaxel (p = 0.004) treated groups. Tumor volumes in the honokiol (p = 0.1942) treated group and paclitaxel (p = 0.9165) treated group showed no significant difference when compared with control. (**b**) Body weights of mice in all groups. The body weights of mice in all four groups were similar. (**c**) Representative mouse from each group. (**d**) Histopathologic analyses of major organs (liver, spleen, kidney, heart and lung) from control and combination treatment group. (**e**) Effects of combination treatment of honokiol and paclitaxel on cell division, proliferation, and apoptosis in vivo. Paraffin-embedded tissue sections from different treatment groups were immunostained with anti-KI-67 for cell proliferation, and TUNEL staining for the detection of apoptotic cells. Apoptotic cells are shown in green; DNA counterstained with DAPI in blue (magnification 200x).

We evaluated the antitumor efficacy of honokiol, paclitaxel, and their combination in treating MDR cancer in the KB-8-5 xenograft model. The treatment was continued for 4 weeks. As shown in **Fig. 6a**, at a dose of 20 mg/kg once per week, a 4-week injection of paclitaxel alone did not significantly inhibit the growth of KB-8-5 subcutaneous tumors, when compared to the vehicle control (p = 0.917). Treatment with honokiol alone (50 mg/kg, 3 times per week, via i.p.) slightly suppressed tumor growth, although the difference was not significant (p = 0.194) (**Fig. 6a**). In contrast, the combination treatment dramatically inhibited the growth of KB-8-5 tumors, when compared with all other groups (vs. control: p<0.0001; vs. honokiol: p = 0.036; vs. paclitaxel: p = 0.004). The average tumor volume in each treatment group at the endpoint was 2585.4±510.0 mm^3^ (control), 1810.2±483.2 mm^3^(honokiol), 2591.3±726.2 mm^3^ (paclitaxel), and 573.9±146.1mm3 (combination). Representative mice from each group are shown in (**Fig. 6c**). These results indicate that honokiol could significantly potentiate the antitumor activity of paclitaxel in MDR cancer xenograft tumors.

We evaluated the systemic toxicity of honokiol, paclitaxel and the combination treatment in the KB-8-5 xenograft model. Compared with the control group, the body weights of mice in all three treatment groups were similar, indicating a negligible toxicity under the tested conditions (**Fig. 6b**). Consistently, histopathologic analyses did not find any considerable tissue damage in the major organs (including liver, spleen, kidney, heart and lung) collected from any treatment group, including the combination treatment (**Fig. 6d** **)**.

We further performed IHC analysis to examine the *in vivo* effect of each treatment on the expression of general tumor biomarkers. The combined treatment significantly reduced the tissue level of Ki-67 in KB-8-5 tumors (percentage of Ki-67 positive cells: 3.84±1.10), when compared to that in the control group (11.96±2.86; p<0.001), honokiol group (7.18±2.07; p = 0.027), or paclitaxel group (11.68±1.82; p<0.001) (**Fig. 6e**). Consistently, TUNEL assay revealed a significant increase in apoptotic tumor cells in the tumor tissues from the combination group (7.07±2.11) when compared with that in the control group (1.64±0.17; p<0.001), honokiol group (5.45±1.19; P<0.05) and paclitaxel group (3.97±3.58; P = 0.08) (**Fig. 6e**). These results indicate that honokiol could sensitize MDR cancer cells to paclitaxel through the induction of apoptosis.

## Discussion

Despite some success in transiently controlling the clinical symptoms of cancer with chemotherapy, a significant percentage of patients develop “multidrug resistance”, or MDR, and inevitably progress with no cure. It is urgent to develop new strategies to overcome MDR and improve the efficacy of chemotherapy. In this study, we investigated the potential utility of the natural compound honokiol in the treatment of chemoresistant cancer using preclinical models. We demonstrated that honokiol could effectively induce cell death in cancer cells regardless of their resistance to the chemotherapy drug paclitaxel. Significantly, honokiol markedly increased the *in vivo* efficacy of paclitaxel in inhibiting the growth of MDR cancer in a xenograft model. We further provided molecular evidence supporting that honokiol induced apoptosis and enhanced chemotherapy through the inhibition of EGFR-STAT3 signaling and downregulation of several survival factors, including survivin, Bcl-2 and Mcl-1. These observations indicate that honokiol could be promising in sensitizing cancer cells to chemotherapy and improving paclitaxel efficacy in clinical settings.

EGFR overexpression has been closely associated with tumor progression, therapeutic resistance and poor clinical outcome in head and neck cancer and other cancer types [16], [17], [18], [19]. The EGFR signaling pathway, including its multiple downstream pathways, such as PI3K/AKT, ERK1/2, JAK/STAT3 and mTOR/NF-κB, plays an important role in the regulation of proliferation, survival, migration and chemoresistance in cancer cells. EGFR signaling, therefore, is being actively pursued as a promising target to develop therapeutics for resistant and recurrent head and neck cancer and other cancer type using small molecule inhibitors and antibodies [20], [21]. In this study, we demonstrated that honokiol decreased both the phosphorylation and expression of EGFR, suggesting an inhibition of EGFR signaling that is consistent with previous studies [5]. As expected, the expression and activity of several important components of EGFR signaling, including AKT, ERK and STAT3, were also inhibited in chemoresistant KB cells upon honokiol treatment. A recent study reported that honokiol downregulates heat shock protein (HSP) 90 in breast cancer cells [22]. HSP90 and other chaperone proteins such as HSP70 and HSP40 regulate EGFR degradation. Interruption of the HSP70/HSP90-folding cycle leads to ubiquitinylation and degradation of EGFR [23] [24]. Therefore, it is possible that honokiol promotes EGFR degradation and inhibits EGFR signaling through an HSP90-dependent mechanism.

Several STAT3 target genes, such as survivin, Bcl-2 and Mcl-1, have central roles in the regulation of survival in cancer cells, and their overexpression has been often linked to resistance to therapy (radiation or chemotherapy), aggressive tumor behavior and shortened survival in many types of cancers [5]–[11]. Moreover, earlier studies reported that paclitaxel induces STAT3 activation and stimulates antiapoptotic protein expression in several cell types, which could be an underlying mechanism for the acquired chemoresistance. Consistently, inhibition of STAT3 and its target genes could enhance the therapeutic efficacy of paclitaxel [25], [26]. In this report, we observed that honokiol inhibits the STAT3 signaling pathway and downregulates the expression of survivin, Bcl-2 and Mcl-1. Since these proteins are involved in the regulation of both intrinsic and extrinsic apoptotic signaling, honokiol suppression of their expression could partially explain the potent cytotoxicity of honokiol in both paclitaxel-sensitive and -resistant KB cells. Further, downregulation of these critical anti-apoptotic proteins may provide a molecular explanation for the chemosensitization capability of honokiol in these cells.

The MDR cancer cell lines used in this study, including KB-8-5, KB-C1, and KB-V1, have been found to substantially overexpress the classical MDR gene product P-glycoprotein (P-gp) [14], [27], [28]. P-gp functions as an energy-dependent efflux pump for which paclitaxel is a substrate, whose overexpression has been correlated with MDR in many cancer types. At the transcriptional level, MDR-1 expression is regulated by NF-κB in different cell types, and several studies reported that honokiol inhibits NF-κB activation and downregulates NF- κB targeting genes including P-gp [1], [3], [29]. In our study, we also found that pre-incubation of resistant cell lines with 10 µm/ml of honokiol for 48 h downregulated P-gp expression and enhanced the intracellular accumulation of paclitaxel (data not shown); thus, downregulation of P-gp by honokiol may also contribute to the synergistic effect of honokiol and paclitaxel in the resistant KB cell lines.

In conclusion, we have shown that honokiol can inhibit cell proliferation and induce apoptosis in multi-drug resistant cell lines. Our study also demonstrated that honokiol can synergistically augment the cytotoxicity of paclitaxel by inhibiting the EGFR-STAT3 signaling pathway and downregulating multiple anti-apoptotic proteins. Honokiol, therefore, is a promising agent in the development of novel treatments for drug-resistant cancer, particularly in combination with conventional therapeutic drugs.

Bcl-2 and Mcl-1 are Bcl-2 family member protein.

## Supporting Information

Figure S1 **Honokiol treatment significantly decreases the levels of survivin mRNA.** KB-8-5 cells were treated with 5 µg/ml of honokiol for the indicated times. Total RNA was isolated from each sample and relative quantification of survivin gene expression was performed with reference to GAPDH RNA as an internal standard.
(DOCX)

Table S1 **Combination index (CI) assay confirmed the synergistic effect of combined treatment with honokiol and paclitaxel in reducing the viability of MDR cancer cells.**
(DOCX)

Methods S1 (DOCX)
