# Supplementary material for: Honokiol Enhances Paclitaxel Efficacy in Multi-Drug Resistant Human Cancer Model through the Induction of Apoptosis
Source: PLoS One. 2014 Feb 25;9(2):e86369. doi: 10.1371/journal.pone.0086369 (PMC3934844; doi:10.1371/journal.pone.0086369)
Supplement: Table S1 — Combination index (CI) assay confirmed the synergistic effect of combined treatment with honokiol and paclitaxel in reducing the viability of MDR cancer cells. (DOCX) [file pone.0086369.s002.docx]

**Table S1.** Combination Index derived by CalcuSyn software.

| Cell Line | Honokiol (µg/ml) | Paclitaxel (ng/ml) | Combination Index |
| --- | --- | --- | --- |
| KB-8-5 | 1  2  3  4 | 15  15  15  15 | 0.55  0.69  0.67  0.76 |
| KB-C1 | 1  2  3  4 | 400  400  400  400 | 0.56  0.72  0.83  0.98 |
| KB-V1 | 1  2  3  4 | 2500  2500  2500  2500 | 0.66  0.70  0.88  0.94 |
