# Supplementary material for: Honokiol Enhances Paclitaxel Efficacy in Multi-Drug Resistant Human Cancer Model through the Induction of Apoptosis
Source: PLoS One. 2014 Feb 25;9(2):e86369. doi: 10.1371/journal.pone.0086369 (PMC3934844; doi:10.1371/journal.pone.0086369)
Supplement: Methods S1 — (DOCX) [file pone.0086369.s003.docx]

Supplementary material (online only):

**Honokiol Enhances Paclitaxel Efficacy in Multi-Drug Resistant Human Cancer Model through the Induction of Apoptosis**

Xu Wang^1^, Jonathan J Beitler^2^, Hong Wang^2^, Michael J. Lee^3^, Wen Huang^2^, Lydia Koenig^1^, Sreenivas Nannapaneni^1^, [A. R. M. Ruhul Amin](http://www.jbc.org/search?author1=A.+R.+M.+Ruhul+Amin&sortspec=date&submit=Submit)^1^, Michael Bonner^5^, Hyung Ju C. Shin^4^, Zhuo (Georgia) Chen^1^, Jack L. Arbiser^5^, Dong M. Shin^1†^

^1^Department of Hematology and Medical Oncology, Winship Cancer Institute, Emory University School of Medicine, Atlanta, GA, USA.; ^2^Department of Radiation Oncology and Otolaryngology, Winship Cancer Institute, Emory University School of Medicine, Atlanta, GA, USA.; ^3^Emory College of Arts and Sciences, Atlanta, GA, USA.; ^4^Quest Diagnostics, Atlanta, GA, USA.; ^5^Department of Dermatology, Winship Cancer Institute, Emory University School of Medicine, and Atlanta Veterans Administration Medical Center, Atlanta, GA, USA.

^†^ Author to whom correspondence should be addressed: Dong M. Shin

email: [dmshin@emory.edu](mailto:dmshin@emory.edu)

Winship Cancer Institute, 1365 Clifton Road, Atlanta, GA 30322, USA.

Running Title: Honokiol Reduces Chemoresistance

Key Words: Honokiol, Drug resistance, EGFR, STAT3, **Material and Methods**

***Real-time PCR.*** Total RNA was isolated from PCI 15A cells with RNeasy Mini Kit (Qiagen, Cat. 74104). Complementary DNA (cDNA) was prepared from each sample with the use of the DyNamocDNA Synthesis Kit (Thermo Scientific, Cat. F-470L), according to the manufacturer’s protocol. Comparative quantitative PCR for survivin was performed with40 cycles in triplicate using fluorescence-based real-time detection with the ABI 7500 Fast Real Time PCR System (Applied Biosystems, CA) with Fast SYBR Green Master Mix (Applied Biosystems, Cat. 4385612). All samples were tested for glyceraldehyde-3-phosphate dehydrogenase (GAPDH) DNA for the comparative quantization. The specific primers for survivin are: 5′-ccaccgcatctctacattca-3′ (forward) and 5′-gcactttcttcgcagtttc-3′ (reverse). Relative quantification of survivin gene expression was performed with reference to GAPDH RNA as an internal standard.

***Combination index assay.***The interaction between honokiol and paclitaxel was analyzed using the CalcuSyn software program (Biosoft, Ferguson, MO) which is based on the Chou and Talalay method[[1](#_ENREF_1)]. In brief, cells were seeded in 96-well plates in triplicateat a density of 5x 10^3^ cells per well. Twenty-fourhours later, serial dilutions of the drugs were prepared for honokiol, paclitaxel and their combination based on their IC_50_, and added to the cells. After incubation for 72 h at 37°C and 5% CO_2_, the cells were subjected to SRB assay. Data from the SRB assay were expressed as the fraction of cells with growth affected (FA) in drug-treated *vs.* untreated cells. The CI was calculated using CalcuSyn software. A CI value of >1 is defined as antagonism, equal to 1 as additivity and <1 as synergy. The experiment was repeated 3 times.

**Reference**

1. Chou TC, Talalay P (1984) Quantitative analysis of dose-effect relationships: the combined effects of multiple drugs or enzyme inhibitors. Adv Enzyme Regul 22: 27-55.
